# Rheumatoid Arthritis Portrayal by UK National Newspapers 2011–2016: A Service User - Led Thematic Analysis of Language Used

**DOI:** 10.1186/s41927-018-0013-z

**Published:** 2018-02-20

**Authors:** Andrew Mark Bassett, Savia de Souza, Ruth Williams, Heidi Lempp

**Affiliations:** King’s College London, Weston Education Centre, 10 Cutcombe Road, London, SE5 9RJ UK

**Keywords:** Newspapers, Language, Rheumatoid arthritis, Thematic analysis, PPI

## Abstract

**Background:**

An important source of knowledge, beliefs, and attitudes about illness is the mass media. Research has established the often negative and emotive language utilised by journalists to report on physical and mental long-term illnesses. The limited amount of research on rheumatological conditions has largely focused on the extent of, and/or accuracy of media coverage. This is the first published study to examine systematically the language used by the United Kingdom (UK) popular press to specifically describe rheumatoid arthritis (RA).

**Methods:**

A patient and public involvement (PPI) approach, involving academics and service users with RA, was used to conduct the research. LexisNexis online repository of print media was searched for articles within a defined five year time frame, which included RA in the headline and/or lead paragraph of 15 UK national non-specialist newspapers. Resultant articles were uploaded to NVivo, and a realist perspective aided a thematic analysis of the data set.

**Results:**

A search of LexisNexis produced 413 newspaper articles, of which 147 met the inclusion criteria. Three themes emerged: (1) language used to describe RA; (2) language used to refer to those who live with RA and; (3) language used to report on potential new treatments for RA. Negative and emotive terms such as ‘attack’, ‘painful’, ‘crippling’, and ‘agony’ were the most frequently used to describe the experience of RA. People diagnosed with RA were often portrayed as ‘sufferers’ or ‘victims’, though neutral language was also deployed. ‘Hope’ and ‘breakthrough’ were the most reported terms for potential new treatments for RA. Across the three themes, tabloid and middle market newspaper articles applied more sensationalised language with attention grabbing headlines and news stories. By contrast, such emotive terminology was less apparent in broadsheets.

**Conclusions:**

The media is a source of information about RA for the general population, but the quality of newspaper journalism about the condition requires improvement. The findings may act as a stimulus for a national public awareness initiative and/or social marketing campaign. How the language currently constructed to describe RA in the press is received by people with RA would be an important area for future research.

## Background

### The representation of illness and disease by the mass media

Lay beliefs about the nature, causes, ramifications, time frame, and terminology of a given illness not only originate from personal experience and the medical care system, but also the mass media and wider culture [1, 2]. The mass media can be defined as “outlets of information and entertainment” [3] that includes television, cinema, newspapers, magazines, and the internet. Recent statistics from the United Kingdom (UK) media regulator, ‘The Office of Communications’ (Ofcom) [4], showed that 94% of the British adult population (defined as aged 16+) followed the news and 29% of them still read newspapers in printed form. Given its important influence, various academic studies have explored the media’s portrayal of long-term health conditions as diverse as eating disorders [5], fibromyalgia [6], chronic fatigue syndrome [7, 8], epilepsy [9, 10], diabetes [11, 12], chronic kidney disease [13], oral cancer [14], gout [15], obesity [16], mental illness [17], and neurological disorders [18].

The language utilised by the mass media in the communication of health issues has the potential to affect the public’s attitudes and perceptions [11]. This recognition has given rise to research, which has examined the media’s usage of language in the representation of long-term conditions. For instance, the media’s consistent application of pejorative terminology to describe mental illness has been demonstrated in numerous studies [17, 19, 20]. An analysis [21] of newspaper clippings found that almost half of British tabloid articles applied derogatory terms, such as ‘nutter’ and ‘looney’ when writing about people with mental health problems. There also has been an overwhelming media fixation on the language of violence in the depiction of schizophrenia, often playing on stereotypes of ‘dangerousness’ and ‘criminality’ [22, 23]. The ‘helpless’ or ‘incompetent’ victim are other demeaning terms written by journalists to characterise people with mental health conditions [24]. Corrigan et al. [25] suggested that this wilful adoption of negative language constituted a form of structural discrimination. Graham Thornicroft [26] asked the question of why the media has tended to trade in the use of such negative stereotypes? One of the main reasons he argues is that as most of the media industry is commercially and profit driven, it has had clear incentives to play on the prejudices of its consumers [26].

Diabetes mellitus also has received negative press coverage [11]. An Australian study [11] showed that terms, such as ‘sufferer’ and ‘diabetic’, which should be avoided when referring to people with diabetes, were still commonly used in news articles. Similarly, research [10] in the popular press in the United States (US) found that despite the protestations of epilepsy organisations, the term ‘epileptic’ featured in nearly half of stories analysed. In addition, some of the articles used mystical or supernatural references to describe seizures.

### Mass media portrayals of rheumatic conditions

Only a few studies have been conducted on the depiction of rheumatological conditions in the mass media. A telephone survey [27] in the US indicated that the media was the most important source of information for the general public about RA. Television (75%), magazines (62%), and newspapers (52%) were all cited by respondents. Other studies have focused on the amount of coverage and/or accuracy of media portrayals of rheumatological conditions in general, rather than RA in particular. For example, early North American research [28] on television news reporting of long-term conditions over an 11 year period, found that there were only 23 news segments dedicated to rheumatological illnesses. This compared to 215 news features on heart disease and 925 on cancer. The study authors’ concluded that the coverage was biased towards diseases with the greatest mortality rates, rather than highly prevalent, but underreported long-term illnesses. A later Dutch study [29] of newspapers, magazines, and medical related television programmes showed that rheumatological conditions were underrepresented in these media outlets. Journalists also emphasised lifestyle factors and alternative treatments. Such a portrayal does not only downplay the seriousness of these conditions, but can lead to a misperception that they could be mitigated or ‘cured’ by will and effort.

Research in the US [30], Mexico [31], and Argentina [32] have confirmed the earlier findings [28, 29] that despite musculoskeletal conditions being commonly prevalent in middle and later life [33], because of their low mortality, they have not inspired much journalistic coverage. Shaw [34] suggested this demonstrates that long-term illnesses (such as RA) do not have media appeal; or to put it more colloquially, “death makes news” [30]. None of the above studies were conducted in the UK, or investigated the usage of language in media depictions of RA or its treatment.

Three recent studies have gone beyond reporting the frequency and/or accuracy of media accounts of rheumatological diseases. The first study [35], an unpublished PhD, used multiple methods of qualitative analyses to investigate how large circulation UK newspapers and magazines conceptualised osteoarthritis (OA). OA was constructed as a multiple phenomenon, as a ‘disease’, ‘condition’, or ‘ailment’. This in turn, was influenced by cultural conceptions of disability-'saints’ or ‘scroungers’- and ageing-'peril’ or ‘promise’, and their media representation was determined by the targeting of readership, financial priorities, and newsworthiness. The study author concluded that media reporting of OA could de-prioritise the condition, and affect societal perceptions about its legitimateness. The second study [15], involved a content analysis of UK and US newspaper articles about gout. It highlighted that humorous comments were often made about gout, linking it to causal beliefs about lifestyle and the consumption of alcohol. The third study by Hanson et al. [36] concentrated on a specific area of media reporting, namely, local and national UK newspaper stories about research on new treatments for rheumatoid arthritis. With very few exceptions, the media narrative was overwhelmingly positive. Three fundamental aspects framed newspaper stories about new medical treatments for RA: “an ‘innovation’ offers ‘hope’ in the context of ‘burden’” [36].

Despite the insights of these three studies [15, 35, 36], to our knowledge there have been no publications on the construction of RA in the UK popular press. Given the prevalence of RA in the UK population [37] and the important role of the media as a source of information about the condition [27], this omission is surprising. In contrast to Hanson et al.*’s* [36] study, our research was led by a researcher with RA, and in addition, was designed to examine the use of language by the printed press in the portrayal of RA. Specifically, the aim of the study was to explore the language used by UK national newspapers in its reporting of rheumatoid arthritis (RA) over a defined five year period between 26th July 2011 and 26th July 2016.

## Methods

### A patient and public involvement (PPI) approach

The study was based on a patient and public involvement (PPI) approach [38] and involved academics and people with RA working together on this project. An inherent advantage of PPI is that it draws on the invaluable perspective of people with RA, who by their daily lived experience of the condition are experts in their own right. PPI can have different degrees of service user involvement in research, including consultation (researchers ask for views/advice from service users and/or carers), collaboration/co-production (researchers and service users and/or carers work together, e.g. to identify research questions), and user-led/controlled (service users make the decisions about the research, e.g. are the principal investigator) [38].

This study was service user-led by the second author, who had primary responsibility for the data collection and analysis of findings. Lempp [38] has argued that PPI is both ethically imperative and important in ensuring research is directly relevant to people with RA and carers. Indeed, the original impetus for the study was the frustration of service users with RA, who we come into daily contact with as a rheumatology department about society’s lack of awareness of RA and its impact on people with the disease. Recent research has shown that members of the public with no direct experience of RA had a limited understanding of the condition [2, 39–41], including a conflation of RA with OA or osteoporosis [39, 41], and a mistaken assumption that RA is a natural consequence of ageing [39, 41].

### Data selection/collection

The decision to restrict the study to newspapers is that they are a highly accessible outlet of media, and they are not limited by the subtopics that they can cover about a given health issue [30]. As Adelman and Verbrugge [30] argued, “scientific findings, therapeutic regimens, government policies and programs, personal stories of adaptation, deaths of individuals, and more” have all featured in the popular press.

The LexisNexis professional electronic newspaper database was searched for articles from national and non-specialist UK newspapers. The tabloid, middle market, and broadsheet newspapers included in this study with their circulation figures in brackets are shown in Table 1.

**Table 1:** Newspapers included in the study

Tabloid/Mass Market	Middle Market	Broadsheet/Quality Press
The Sun (1,666,715) ^#^ Daily Mirror (724,888) ^#^ Daily Star (443,452) ^#^ The People (240,846) ^#^	Daily Mail (1,511,357) ^#^ Daily Express (392,526) ^#^ The Mail on Sunday (168,164) ^#^	Daily Telegraph (472,258) ^#^ The Times (451,261) ^#^ The Guardian (156,756) ^#^ The Independent (55,193) ^*^ The i (266,768) ^#^ The Sunday Times (792,324) ^#^ The Sunday Telegraph (359,400) ^#^ The Observer (185,752) ^#^

#Figures shown by average circulation for January 2017 [42]

*No figures are available for the Independent broadsheet newspaper for January 2017, as publication ceased in print form, and it is now only available online. The stated figure is therefore based on average circulation for January 2016 [43] when it was still in print

Tabloids can be defined by their straightforward use of language and “light, bright, and vivid writing, shorter stories, and extensive use of photographs and graphics” [44], that appeal to a wide readership. In addition, the colourful imagery that is typical of a tabloid’s front page is anchored by striking headlines [44]. Another feature of these mass market newspapers is the subject matter of entertainment, sports, crime, and celebrity stories that often invoke scandal or have a sexual element [44]. Although these characteristics are also found in the broadsheet newspapers, they assume greater importance and prominence in the tabloids [44].

By contrast, broadsheets also known as quality newspapers were traditionally defined by their large format and their higher level of language compared to tabloids [45]. Furthermore, they are characterised by an editorial and presentational style where the focus is on the ‘serious news’ of politics, business, economics, and world affairs [46]. The middle market newspapers in contrast, combine the entertainment features of the tabloids with the coverage of ‘serious’ topics associated with the quality press [47].

The inclusion criteria for the analysis were: (i) articles with the phrase ‘rheumatoid arthritis’ in the headline and/or lead paragraph, and (ii) published between 26th July 2011 and 26th July 2016. The term rheumatoid arthritis (RA) refers to "a chronic inflammatory disease characterized by joint swelling, joint tenderness, and destruction of synovial joints, leading to severe disability" [48]. As one person (second author) had primary responsibility for the analysis of the newspaper items, a five year time span for the identification of articles was agreed among the authors as manageable given the amount of data. A 5 year time period has also been deemed appropriate in other research [15, 17] on newspaper portrayals of long term illnesses. The exclusion criteria were: (i) items which referred to RA only once, (ii) duplicate articles, (iii) letters that sought medical advice, and (iv) product advertisements.

### Data analysis

All articles were uploaded to NVivo Pro 11 [49] to aid the analytical process. A thematic analysis based on a realist perspective [50] was applied to the dataset of newspaper articles. In this approach, codes and themes were not predetermined deductively by a theoretical framework, but were generated inductively from the data [51]. As the results of the literature search/review discovered little information about the language used in the popular press to portray RA, an inductive orientation [51] to the analysis fitted in with the explorative nature of the research.

In reference to a grounded theory approach, the second author systematically interrogated the data set through ‘open coding’ [52] with the ‘constant comparative’ method [53]. In this way, simultaneous comparison of codes and data were identified, followed by the refinement of codes into themes. A random sample of 25% of the coded data was then cross-checked by the third author and a consensus was reached on final codes and themes. This form of triangulation [54] was designed to limit researcher subjectivity during the analytical process. The results of the thematic analysis were member checked [55] by proxy with a group of clinicians (e.g. nurses and doctors) drawn from the authors’ academic rheumatology department. We used this strategy, because departmental expert service users recommended not to approach service users for the validation of findings, but instead involve clinicians in this process. Service users have at times disclosed negative reactions to media coverage of RA to clinical staff during consultations.

## Results

The initial search in the LexisNexis database generated 413 articles from 15 national and non-specialist newspapers, of which 147 qualified according to the inclusion criteria. Table 2 provides a breakdown of the frequency of articles about RA by newspaper source.

**Table 2:** Frequency of Articles about Rheumatoid Arthritis (RA) by Newspaper Source

Newspaper Source	Number of Articles about Rheumatoid Arthritis (RA) (*n=*)
Daily Express	31
Daily Mail	28
Daily Mirror	27
Daily Telegraph	23
The Sun	13
The Times	5
The Guardian	4
The Sunday Times	3
Daily Star	3
Sunday Telegraph	2
Mail on Sunday	2
The i	2
The Independent	2
The People	2
The Observer	0
Total	147

The majority (*n* = 106, 72%) appeared in tabloid/middle market newspapers, with just 28% (*n* = 41) featured in broadsheets. 81% (*n* = 86) of tabloid/middle market articles were printed in the Daily Express, Daily Mail, and Daily Mirror. 56% (*n* = 23) of broadsheet items were in the Daily Telegraph alone.

Three themes were identified in the thematic analysis, which conveyed how UK national newspapers used language to report about RA. Themes included: (1) language used to describe RA; (2) language used to refer to those who live with RA; and (3) language used to report on potential new treatments for RA. A table is presented for each of the themes and each table includes the following headings: (i) the names of the codes listed under each theme; (ii) the number and proportion of newspaper articles a particular code is cited in; (iii) the number of references to a code across the whole dataset of newspaper items; (iv) the number of references to a code by tabloid/middle market newspapers or broadsheets and; (v) the newspapers with the highest number of references for a given code. All the descriptive statistics stated in each of the three thematic tables were derived by simple counting methods [56]. Quotations from newspaper articles that met the inclusion criteria are reported to illustrate the themes. The letter case presentational style of the original articles is kept. Many of the newspaper headlines capitalised all letters, and some of the articles used a combination of upper and lower case letters.

### Theme 1: Language used to describe rheumatoid arthritis (RA)

Table 3 indicates the language used by UK national newspapers to describe RA.‘Attack’ was the most cited term across the newspaper dataset to refer to the experience of RA, had the highest proportion and number of articles, and was more likely to be used in the tabloid or middle market press than the broadsheets. The Daily Express and the Daily Mail, both of which are middle market newspapers, contributed the highest number of references to ‘attack’ to describe RA. Indeed, the Daily Express alone had over half of the references to the word ‘attack’ in the dataset.

“Rheumatoid arthritis occurs when the immune system attacks the joints, affects about 700,000”. (Daily Express, 16 February 2013)

“Rheumatoid arthritis, which is one of the most common forms of the disease, occurs when the immune system attacks the joints by mistake”. (Daily Mail, 31 August 2015)

**Table 3:** Coding Scheme for Theme 1

Code	Number of Articles [*n* = (%)]*	Number of References [*n*=]	The number of references by tabloid/middle market or broadsheet [*n* = (%)]	Newspapers with the highest number of references [*n* = (%)]
Attack	38 (26)	49	Tabloid/Middle Market 39 (80) Broadsheet 10 (20)	Daily Express 29 (59) Daily Mail 17 (35)
Painful	33 (22)	47	Tabloid/Middle Market 41 (87) Broadsheet 6 (13)	Daily Mirror 18 (38) Daily Express 11 (23)
Crippling	29 (20)	33	Tabloid/Middle Market 32 (97) Broadsheet 1 (3)	Daily Express 20 (61) Daily Mirror 6 (18)
Agony	25 (17)	32	Tabloid/Middle Market 30 (94) Broadsheet 2 (6)	Daily Express 21 (66) Daily Mail 3 (9) Daily Mirror 3 (9)
Incurable	24 (16)	31	Tabloid/Middle Market 28 (90) Broadsheet 3 (10)	Daily Express 14 (45) Daily Mirror 7 (23)
Destroying	12 (8)	18	Tabloid/Middle Market 16 (89) Broadsheet 2 (11)	Daily Express 11 (61) Daily Mirror 3 (17)
Debilitating	11 (7)	12	Tabloid/Middle Market 10 (83) Broadsheet 2 (17)	Daily Express 5 (42) Daily Mail 3 (25)
Misery	8 (5)	8	Tabloid/Middle Market 7 (87) Broadsheet 1 (13)	Daily Express 5 (62) Daily Mirror 2 (25)
Struggle	7 (5)	10	Tabloid/Middle Market 10 (100) Broadsheet 0 (0)	Daily Mirror 4 (10) Daily Mail 3 (10)
Battle	3 (2)	5	Tabloid/Middle Market 3 (60) Broadsheet 2 (40)	Mail on Sunday 2 (40) Sunday Telegraph 2 (40)
Excruciating	3 (2)	4	Tabloid/Middle Market 4 (100) Broadsheet 0 (0)	Daily Mirror 4 (100)
Ravaging	2 (1)	2	Tabloid/Middle Market 1 (50) Broadsheet 1 (50)	Mail on Sunday 1 (50) Sunday Telegraph 1 (50)
Devastating	2 (1)	2	Tabloid/Middle Market 2 (100) Broadsheet 0 (0)	Daily Express 2 (100)
Unrelenting	1 (1)	1	Tabloid/Middle Market 1 (100) Broadsheet 0 (0)	Daily Mail 1 (100)
Scourge	1 (1)	1	Tabloid/Middle Market 1 (100) Broadsheet 0 (0)	Daily Mirror 1 (100)

*Percentage calculated where total number of articles analysed *n* = 147

‘Painful’ was also a word frequently used by journalists in the context of RA. This term was most referenced in the Daily Mirror and the Daily Express, and was far more common in tabloid and middle market newspapers than the quality press. The following example featured in the headline of a tabloid.

“[RA] ARTHRITIS IS TOO PAINFUL TO ENJOY LIFE”. (Daily Mirror, 24 January 2012)

Another illustration was the headline of a features section in the Daily Mirror.

“MY HANDS ARE GNARLY AND PAINFUL, BUT I WON'T LET [RA] ARTHRITIS TAKE OVER”. (Daily Mirror, 3 April 2012)

Just 1 (3%) mention of the word ‘crippling’ to convey the experience of RA was in the broadsheet press. All other references to this term were in tabloid or middle market newspapers. The Daily Express alone had over half of the mentions to ‘crippling’. The following was a headline from a news section.

“BREAST-feed your BaBy and ward off crippling [RA] ARTHRITIS”. (Daily Express, 7 January 2014)

Under this headline, the journalist made the claim that:

“Scientists found that [breastfeeding] mothers were around 50 per cent less likely to develop the crippling joint condition compared with those who never breast-fed”. (Daily Express, 7th January 2014)

The language of ‘agony’ and ‘incurable’ was also applied in newspaper portrayals of RA. Again, these descriptors for RA were far more common in tabloid or middle market newspapers, such as the Daily Express, Daily Mail, and Daily Mirror.

“hundreds of thousands of Britons who suffer the agony of [RA] arthritis”. (Daily Mail, 4 June 2015)

“Nearly 10 million people in Britain are blighted by incurable [RA] arthritis”. (Daily Express, 16 February 2013)

### Theme 2: Language used to refer to those who live with rheumatoid arthritis (RA)

Table 4 shows the type of language that was used by the UK popular press to describe those who live with RA. The term ‘sufferers’ had the most number of references across the entire dataset of newspaper articles, and in addition, was more likely to be found in the tabloid/middle market press than in broadsheets. Over half (58%) of the mentions of ‘sufferers’ were in the Daily Express and Daily Mirror. The following was from a headline from the latter newspaper.

“Isolation of arthritis [RA] sufferers”. (Daily Mirror, 11 June 2013)

**Table 4:** Coding Scheme for Theme 2

Code	Number of Articles [*n* = (%)]*	Number of References [*n*=]	The number of references by tabloid/middle market or broadsheet [*n* = (%)]	Newspapers with the highest number of references [*n* = (%)]
Sufferers	68 (46)	117	Tabloid/Middle Market 92 (79) Broadsheet 25 (21)	Daily Express 39 (33) Daily Mirror 29 (25)
Patients	50 (34)	123	Tabloid/Middle Market 91 (74) Broadsheet 32 (26)	Daily Express 43 (35) Daily Mail 32 (26)
People	30 (20)	41	Tabloid/Middle Market 27 (66) Broadsheet 14 (34)	Daily Express 12 (29) Daily Mirror 9 (22) Telegraph 9 (22)
Victims	5 (3)	6	Tabloid/Middle Market 5 (83) Broadsheet 1 (17)	Daily Mail 3 (50) Daily Express 2 (33)
Copers	4 (3)	4	Tabloid/Middle Market 4 (100) Broadsheet 0 (0)	Daily Express 2 (50)
Individuals	3 (2)	3	Tabloid/Middle Market 3 (100) Broadsheet 0 (0)	Daily Mirror 1 (33) Daily Mail 1 (33) Daily Express 1 (33)
Brave	2 (1)	2	Tabloid/Middle Market 2 (100) Broadsheet 0 (0)	Daily Mirror 1 (50) Daily Star 1 (50)
Arthritics	1 (1)	1	Tabloid/Middle Market 0 (0) Broadsheet 1 (100)	Telegraph 1 (100)

*Percentage calculated where total number of articles analysed *n* = 147

The next example, was from the news section of the Daily Express.

“many [RA] sufferers are forced to have joint replacement surgery”. (Daily Express, 28 March 2015)

Another term was ‘victim’, which tabloid journalists in particular, used for those with RA. The following was a headline from the Daily Mail.

“NHS GREEN LIGHT FOR DRUG THAT COULD BRING RELIEF TO 40,000 [RA] ARTHRITIS VICTIMS”. (Daily Mail, 22 February 2012)

The term ‘victim’ was again, found in this Daily Mail article.

“Half of victims are unable to work through disability [with RA] within ten years”. [Daily Mail, 22 February 2012).

However, the more neutral terms of ‘patients’ and ‘people’ were also applied frequently in the popular press to refer to those with RA. Although represented more in the tabloid and middle market newspapers, around a third of references to ‘people’ or the singular ‘person’ was in the quality press.

“A person with [RA] chronic pain can become trapped in a downward spiral-pain destroys sleep, and then without sleep the body cannot stay healthy”. (The Guardian, 27 June 2016)

### Theme 3: Language used to report on potential new treatments for rheumatoid arthritis (RA)

The final theme that emerged from the analysis as shown in Table 5, was concerned with the language that the popular press drew upon to report potential new drugs or medical technologies for RA. In many cases, journalists used the language of ‘hope’ to frame the discussion of potential treatments for the condition. The term ‘hope’ was more frequent in the tabloid and middle market newspapers, with 65% of references in the Daily Express and Daily Mirror alone. Under the headline, “Daily pill that could end arthritis agony in a week”, a news section of the Daily Express made the following claim.

“A POWERFUL compound has been discovered that dramatically reduces joint inflammation and brings hope for hundreds of thousands of [rheumatoid] arthritis sufferers... The SR2211 compound, tested successfully on mice, also significantly cuts bone and cartilage erosion”. (Daily Express, 28 December 2013)

**Table 5:** Coding Scheme for Theme 3

Code	Number of Articles [*n* = (%)]*	Number of References [*n*=]	The number of references by tabloid/middle market or broadsheet [*n* = (%)]	Newspapers with the highest number of references [*n* = (%)]
Hope	30 (20)	43	Tabloid/Middle Market 34 (79) Broadsheet 9 (21)	Daily Express 18 (42) Daily Mirror 10 (23)
Breakthrough	21 (14)	31	Tabloid/Middle Market 29 (94) Broadsheet 2 (6)	Daily Express 18 (58) Daily Mirror 8 (26)
Groundbreaking	7 (5)	7	Tabloid/Middle Market 5 (71) Broadsheet 2 (29)	Daily Express 4 (57) Telegraph 2 (29)
Cure	2 (1)	3	Tabloid/Middle Market 3 (100) Broadsheet 0 (0)	Daily Mirror 2 (75) Daily Express 1 (25)
Miracle	1 (1)	1	Tabloid/Middle Market 1 (100) Broadsheet 0 (0)	Daily Mirror 1 (100)

*Percentage calculated where total number of articles analysed *n* = 147

The next example discusses the potential emergence of treatment for RA, not only through the language of ‘hope’, but also ‘cure’.

“A TINY electric implant can end the crippling pain of rheumatoid arthritis, scientists have revealed. It brings hope of a cure to 400,000 UK sufferers and millions worldwide”. (Daily Star, 23 December 2014)

‘Breakthrough’ was another frequent term used by newspaper journalists to describe the potential positive impact of new treatments for RA. However, the language of ‘breakthrough’ was much more likely to be found in the tabloid or middle market press. 84% of references to this term were in the Daily Express and Daily Mirror.

“A major breakthrough could end the misery of [rheumatoid] arthritis by helping to create an entirely new class of drugs to treat the disease... Researchers found a way to block cells in the body which directly attack the cartilage, causing irreparable damage to affected joints”. (Daily Express, 21 May 2015)

“Experts discovered antibody drug tocilizumab is almost four times more likely to halt progression of the condition [rheumatoid arthritis] than the most widely-prescribed alternative...the potentially life changing breakthrough has been welcomed by almost one million arthritis sufferers...”. (Daily Mirror, 9 June 2012)

## Discussion

The resultant newspaper articles tended to draw on negative and emotionally laden language to convey the experience of RA. However, this was more apparent in the tabloid or middle market newspapers than in the broadsheets. Neutral terms, such as ‘people’ and ‘individuals’ were used to describe those living with RA. However, they often were viewed by the popular press in a passive sense, as ‘sufferers’ or ‘victims’. The media’s application of language in the context of potential new medical technologies or drugs for RA, overwhelmingly stressed their positive effects. This is despite the fact that much of the scientific research highlighted in the printed press over the five year time frame of this study was still in development and often untested in humans.

Terms such as ‘attack’, ‘destroying’, ‘struggle’, and ‘battle’, which were readily written by journalists in reference to RA, seemed to draw on metaphors of warfare [57–59]. Lakoff and Johnson [60] have suggested that conceptual systems are designed to function in a metaphorical sense. That is, metaphors allow us to communicate concepts that are abstract or complex in simplified terms [60]. Metaphoric language has a long and durable history in medicine [57–59, 61]. Metaphors of transgression and punishment have been commonly used in tuberculosis (TB) research and clinical practice [61], and military metaphors are a noticeable feature of discourses around the medical treatment of cancer [57–59]. The use of militaristic metaphors in lay (media) or medical discourse can create a passive perception of the patient and a narrow focus with the biomedical aspects of the disease [58]. Thus, other parts of the illness experience, and especially those that are of significance to the person dealing with the illness, such as the social and psychological impact, are disregarded [58].

The emotive vocabulary that we found in the printed press portrayal of RA accorded with the findings of Hanson et al*’s* [36] study, which showed the negative newspaper representation of the ‘burden’ of RA through its use of terms such as “‘crippling’, ‘debilitating’ and ‘constant agony’”. However, in our analysis, we discovered differences in newspaper type, with the tabloid and middle market press far more likely to have drawn on negative terminology to describe RA than the broadsheets. The boundaries between information and entertainment known as ‘infotainment’ [62] are often blurred in the tabloid and middle market newspapers, where information is mixed “with strategies of shock and titillation” [62], in order to maximise the consumption of news. However, the more ‘serious’ editorial policy of the broadsheets, means that in the quality press, there is typically a move away from ‘infotainment’ towards the communication of factual information [62]. It was also evident that our findings aligned with the observation from Hanson et al*’s* [36] research, in how the printed press deployed startling statistics, such as “700,000 sufferers” or “1000 a year have to give up work” [36], to associate RA with a societal ‘burden’ or ‘cost’.

Research has analysed the media’s usage of language in the context of people with mental health problems [17, 19–23], epilepsy [9, 10], diabetes [11], oral cancer [14], and the rheumatological conditions of gout [15] and osteoarthritis [35]. However, our study was the first published one on how the popular press has represented through language those who live with RA. We found that newspapers did use neutral terminology to refer to those living with RA. However, people with RA were also constructed as passive ‘sufferers’ and ‘victims’. In 2011, the organisation ‘Diabetes Australia’ developed a position statement called ‘A new language for diabetes’ [63], which advocated person-first language [64] when speaking about people with diabetes mellitus. It also described in detail, the terms not to be used when talking about diabetes, as well as defining an approved set of terms [63]. For example, when referring to individuals with diabetes, ‘person with diabetes’ or ‘person living with diabetes’ is preferred language to use than ‘diabetic’ or ‘sufferer’ [11]. The position statement arose out of the recognition that the appropriate use of language is not just required of those working in the health services, but also the mass media, which is an important source of health information for the general public [11]. We suggest the development of a similar position statement that would recommend person-first language when writing or speaking about RA and the people that live with the condition.

The tabloid and middle market newspapers in particular, drew on the language of ‘hope’ or ‘breakthrough’ to report on potential treatments for RA. This finding confirmed Hanson et al.*’s* study [36], that the popular press have created a uniformly positive narrative about emerging, but still in development, new medical technologies and drugs for RA. However, the media’s overhyping of scientific research is not just specific to RA, but to health conditions in general. UK [65] and international research [66–68] demonstrated that the popular press has underplayed the possible harms and risks of new medical treatments, whilst exaggerating their benefits. It also recalls Dorothy Nelkin’s [69] argument, that journalists in seeking to inform as well as to entertain (‘infotainment’), are attracted to the novel in scientific study, even though such research may only exist in tentative form. We concur with Schwitzer’s [66, 67] claim, that the quality and transparency of health-related journalism would significantly improve by the media giving careful consideration to both the benefits and risks of new medical interventions.

People with RA and patient organisations may utilise the study findings in future educative efforts aimed at journalists. This could also be in conjunction with a national RA public awareness campaign or social marketing with charities. A model example for a nationwide campaign is ‘Time to Change’ (TTC) [70]. TTC is the largest ever initiative in England, which has aimed to improve the public’s understanding of mental health and therefore tackle stigma and discrimination [70]. TTC has involved engaging with communities and various stakeholder groups (e.g. professional membership groups), the dissemination of social marketing information, public relation strategies, and the facilitation of events designed to encourage social contact between people with and without mental health problems [70]. A research evaluation of the impact of TTC’s social marketing interventions found positive changes in terms of greater knowledge of, and more favourable attitudes towards mental illness [71]. The ‘European League Against Rheumatism’ (EULAR), ‘Don’t Delay, Connect Today’ initiative also provides a starting place for a public awareness campaign directed at RA [72]. The initiative seeks to overcome the myths that pervade the public consciousness about RA and to highlight the seriousness of the condition, which may facilitate early diagnosis [72].

A strength of this study is that the data was drawn from a 5 year timescale. In addition, the thematic analysis was based on an extensive range of daily and weekly tabloid/middle market and broadsheet newspapers with different ideological perspectives, and fits in well with the long tradition of media studies of popular press representations of illness. The study analysed textual data and did not analyse other contextual aspects of newspaper representations of RA, such as images. The study did not involve an examination of television, radio, magazines, the internet and local news media, and therefore, the findings cannot be applied to these other means of mass communication. Relevant newspaper articles may have been omitted from the study, as an inclusion criterion was to restrict the search of items with the phrase ‘rheumatoid arthritis’ only to the headline and/or lead paragraph.

We concur with Seale [73], that more research should take an audience reception approach when examining media portrayals of health conditions. In particular, we recommend that future research should explore how the language used to construct RA by newspapers is received and interpreted by people with the condition. We realise there may be sensitive issues with sharing information on how the media has constructed RA. However, service user involvement in such research may be helpful in working towards a position statement of person-first language in RA media reporting.

## Conclusion

This was the first published study to analyse the language used to portray RA in the widely circulating UK national press. During the five year time period looked at for this study, journalists writing for tabloid and middle market newspapers in particular, regularly drew on negative and overly emotive terminology to convey the experience of RA. Although the neutral language of ‘people’ and ‘individuals’ was used to describe those living with RA, the passive terms of ‘sufferer’ and ‘victim’ was also found in newspapers’ discourse. An overwhelming media focus was placed on the positive effects of emerging scientific research for the treatment of RA. Considering that research has shown that much of the general population has a limited knowledge of RA [2, 39–41], and that the media is a primary source of information about the condition [27], the quality of newspaper reporting on RA in the UK needs improvement.

## Abbreviations

OA: Osteoarthritis
Ofcom: The Office of Communications
PPI: A patient and public involvement approach
RA: Rheumatoid Arthritis
TB: Tuberculosis
TTC: Time to Change
UK: United Kingdom
US: United States
